# Early remodelling of the extracellular matrix proteins tenascin‐C and phosphacan in retina and optic nerve of an experimental autoimmune glaucoma model

**DOI:** 10.1111/jcmm.12909

**Published:** 2016-07-04

**Authors:** Sabrina Reinehr, Jacqueline Reinhard, Susanne Wiemann, Gesa Stute, Sandra Kuehn, Julia Woestmann, H. Burkhard Dick, Andreas Faissner, Stephanie C. Joachim

**Affiliations:** ^1^ Experimental Eye Research InstituteUniversity Eye HospitalRuhr‐University BochumBochumGermany; ^2^ Department of Cell Morphology and Molecular NeurobiologyRuhr‐University BochumBochumGermany

**Keywords:** glaucoma, retina, optic nerve, extracellular matrix, tenascin‐C, phosphacan/RPTPβ/ζ, retinal ganglion cells, GFAP

## Abstract

Glaucoma is characterized by the loss of retinal ganglion cells (RGCs) and optic nerve fibres. Previous studies noted fewer RGCs after immunization with ocular antigens at 28 days. It is known that changes in extracellular matrix (ECM) components conduct retina and optic nerve degeneration. Here, we focused on the remodelling of tenascin‐C and phosphacan/receptor protein tyrosine phosphatase β/ζ in an autoimmune glaucoma model. Rats were immunized with optic nerve homogenate (ONA) or S100B protein (S100). Controls received sodium chloride (Co). After 14 days, no changes in RGC number were noted in all groups. An increase in *GFAP* mRNA expression was observed in the S100 group, whereas no alterations were noted *via* immunohistochemistry in both groups. Extracellular matrix remodelling was analyzed after 3, 7, 14 and 28 days. Tenascin‐C and 473HD immunoreactivity in retinae and optic nerves was unaltered in both immunized groups at 3 days. At 7 days, tenascin‐C staining increased in both tissues in the ONA group. Also, in the optic nerves of the S100 group, an intense tenascin‐C staining could be shown. In the retina, an increased tenascin‐C expression was also observed in ONA animals *via* Western blot. 473HD immunoreactivity was elevated in the ONA group in both tissues and in the S100 optic nerves at 7 days. At 14 days, tenascin‐C and 473HD immunoreactivity was up‐regulated in the ONA retinae, whereas phosphacan expression was up‐regulated in both groups. We conclude that remodelling of tenascin‐C and phosphacan occurred shortly after immunization, already before RGC loss. We assume that both ECM molecules represent early indicators of neurodegeneration.

## Introduction

Glaucoma is a neurodegenerative disease that leads to irreversible vision defect and is defined by the loss of retinal ganglion cells (RGCs) and their axons. Its exact pathomechanisms are currently poorly understood. Present therapies tend to lower intraocular pressure (IOP), which is the most important risk factor of this disease. Nevertheless, this can only slow down progression and cannot stop the cell loss. Therefore, it is necessary to analyze the pathomechanisms more precisely.

Recent studies could demonstrate that the immune system plays a key role in glaucoma 1. To investigate the mechanisms of the immune system in more detail, an IOP‐independent animal model, called experimental autoimmune glaucoma (EAG) model, was developed 2. Here, animals were immunized with ocular antigens, namely a bovine optic nerve antigen homogenate (ONA) or with the S100B protein (S100). ONA is a homogenate that contains a mixture of neuronal and glial antigens, whereas S100 is a purified glial protein and a component of the ONA. Increased autoantibody levels against S100 were detected in patients with glaucoma 3. The immunization with ONA and S100 leads to a loss of RGCs and to optic nerve damage, without IOP elevation, starting at about day 28 after immunization 2, 4, 5. Also, autoreactive antibodies were found in the retina and the optic nerve of immunized animals as well as signs of reactive gliosis and apoptosis 6.

A variety of studies indicate that retina as well as optic nerve damage is accompanied by remodelling of extracellular matrix (ECM) proteins. The ECM consists of a meshwork of glycoproteins and proteoglycans. Constituents of the ECM form the milieu surrounding retinal cells, provide structural and mechanical support and regulate retinal homeostasis as well as cellular signalling 7. Interestingly, in patients with primary open‐angle glaucoma, who display IOP elevation, an up‐regulation of the ECM protein tenascin‐C was observed in the optic nerve head 8. Similar results were noted in an ocular hypertension animal model 9. Also, the importance of matricellular proteins in the trabecular meshwork (TM) during high‐tension glaucoma is well documented 10. Nevertheless, until now, no studies exist that report ECM remodelling in an IOP‐independent glaucoma animal model.

The aim of this study was to analyze ECM remodelling in the neurodegenerative retina and optic nerve of a pressure‐independent EAG model.

As a result of the described importance of phosphacan and tenascin‐C during retinal development and in central nervous system (CNS) regeneration, we focused on the potential role of these ECM constituents in the EAG model.

The glial‐derived chondroitin sulphate proteoglycan (CSPG) phosphacan, also called DSD‐1‐PG 11, is a secreted splice variant of the receptor protein tyrosine phosphatase (RPTP)‐β/ζ 12. This receptor is expressed in two splice variants, namely RPTPβ/ζ_long_ and RPTPβ/ζ_short_, both harbour two intracellular protein tyrosine phosphatase (PTP) domains. Phosphacan and RPTPβ/ζ_long_ carry the DSD‐1 epitope, a glycosaminoglycan modification recognized by the 473HD antibody. In the retina, phosphacan is initially detectable at embryonic day 13, peaks around post‐natal day 6 and is progressively down‐regulated until adolescence, where the molecule is restricted to Müller glia cells 7, 13. RPTPβ/ζ_long_ exhibits minor expression in the adult retina 13. Therefore, in this study, 473HD immunoreactivity can be referred to as phosphacan.

Tenascin‐C represents a hexameric multi‐modular glycoprotein that displays diverse cellular functions in the CNS, including adhesion, axon growth, migration, proliferation and synaptic plasticity 7, 14, 15, 16. Each of its six chains is composed of several epidermal growth factor‐like repeats as well as a variable number of fibronectin type III–like domains, as a result of alternative splicing 14, 17. Tenascin‐C directly interacts with other ECM components, including the CSPGs neurocan 18 and phosphacan 19, 20 and integrin receptors 21. In addition, tenascin‐C is cleaved by various matrix metalloproteinases (MMPs) 22. In the retina, tenascin‐C is restricted to the inner and outer plexiform as well as nerve fibre layer. In the optic nerve, tenascin‐C is expressed by astrocytes 7. Glia‐enriched tenascin‐C displays prominent expression during early CNS development. Later on, it is down‐regulated, but re‐expressed, *e.g*. after brain injury 23, 24. Both molecules were examined through immunohistochemistry, Western blot analysis and quantitative real‐time PCR (qRT‐PCR) at several points in time following immunization.

## Materials and methods

### Animals

All experiments involving animals were approved by the animal care committee of North Rhine‐Westphalia (Germany). The animals were treated according to the Association for Research in Vision and Ophthalmology statement for the use of animals in ophthalmic and vision research and the European Union regulations for the use of animals in research. The animals were maintained under a 12 hrs light/12 hrs dark cycle in a temperature‐controlled animal facility. Food and water were available *ad libitum*. All animals underwent regular ophthalmic and neurological examinations.

### Experimental groups

Six‐week‐old male Lewis rats (Charles River, Sulzfeld, Germany) were randomized into three groups: ONA, S100 and control animals (Co). The animals were sacrified after 3, 7, 14 and 28 days post‐immunization and eyes and optic nerves were harvested and processed further.

### Immunization

The preparation and immunization of ONA were performed as previously described 2, 5. For ONA preparation, fresh bovine eyes were obtained from the local abattoir. The optic nerves were dissected behind the optic nerve head and the dura mater was removed. The tissue was transferred to a cooled mortar and was grounded until it was a pulverized texture. The powder was suspended in PBS and a concentration of 8 mg/ml was set. The S100 antigen is commercially available (Sigma‐Aldrich, Munich, Germany) 4.

Rats received either 8 mg/ml ONA or 1 mg/ml S100 protein intraperitoneally. The antigens were mixed with incomplete Freund's adjuvant (500 μl in ONA, 200 μl in S100) and 3 μg pertussis toxin (both Sigma‐Aldrich). Control animals were injected with NaCl in Freund's adjuvant and pertussis toxin.

### Tissue preparation for histological analysis

Eyes and optic nerves were obtained 3, 7, 14 and 28 days after immunization from all groups, fixed in 4% PFA, dehydrated in sucrose and embedded in Tissue‐Tek^®^ (Thermo Fisher Scientific, Waltham, MA, USA). Cross‐sections of the retinae (10 μm) and longitudinal sections of the optic nerves (4 μm) were cut with a Cryostat (Thermo Fisher Scientific) and mounted onto Superfrost Plus slides (Thermo Fisher Scientific).

### RGC evaluation

14 days after immunization, retinal cross‐sections (*n* = 5/group) were suspended in 10% donkey serum in 0.1% Triton‐X100 in PBS for 60 min. A goat polyclonal Brn‐3a antibody (1:100; Santa Cruz, Heidelberg, Germany) was incubated overnight. Brn‐3a is an established marker for RGCs 25, 26. Incubation of the secondary antibody donkey anti‐goat Alexa 488 (1:500; Dianova, Hamburg, Germany) was performed for 60 min. the next day. Nuclear staining with 4′,6 diamidino‐2‐phenylindole (DAPI; Serva Elektrophoresis, Heidelberg, Germany) was included to facilitate orientation on the slides. Negative controls were performed by using the secondary antibody only. The photographs were taken using a fluorescence microscope (Axio Imager M1; Zeiss, Göttingen, Germany). Each time, two photos of the peripheral and two of the central part of each section were captured. The images were transferred to Corel Paint Shop Pro (V13; Corel Corporation, Ottawa, Canada) and excerpts were cut out, including the GCL, to get the same area of each retina. RGCs were counted using ImageJ software (V. 1.47; NIH, Bethesda, MD, USA).

### Analysis of ECM components

The evaluation of the immunoreactivity of ECM components was performed 3, 7, 14 and 28 days after immunization. Retinae and optic nerves (*n* = 4–6/group/point in time) were treated with 3% goat serum, 1% bovine serum albumin and 0.5% Triton‐X 100 in PBS for 60 min. For tenascin‐C labelling, an anti‐tenascin‐C antibody 27 (1:200) was used. Staining of phosphacan/RPTPβ/ζ_long_ was performed with an anti‐473HD antibody 28 (1:200). Both primary antibodies were incubated overnight (Table 1). The complementary secondary antibodies goat anti‐rabbit Cy3 (1:250; Dianova) and goat anti‐rat Cy2 (1:250; Dianova) were incubated the next day for 2 hrs. Nuclear staining with TO‐PRO‐3 (1:400; Invitrogen, Darmstadt, Germany) was added to visualize all cell nuclei. Images were taken with a confocal laser‐scanning microscope LSM 510 META (Zeiss). Laser lines and emission filters were optimized with the Zeiss LSM Image Browser software 29, 30. Tenascin‐C and 473HD immunoreactivity were analyzed using an ImageJ macro 6. Briefly, we transformed the images into greyscale and after background subtraction the lower and upper thresholds were set (Table 2). The percentage of the labelled tenascin‐C^+^ and 473HD^+^ areas was measured for each picture using the macro. These values were added in a macro and the area fraction (%) was calculated automatically. The measured area fraction (%) for each picture is given in a table afterwards 31.

**Table 1:** Primary and secondary antibodies applied for immunohistochemistry of retinal and optic nerve tissue

Primary antibodies					Secondary antibodies			
Antibody	Company	Clone	Tissue	Dilution	Antibody	Company	Tissue	Dilution
Brn‐3a	Santa Cruz	C‐20	Retina	1:100	Donkey anti‐goat Alexa Fluor 488	Dianova	Retina	1:500
GFAP	Millipore	Polyclonal	Retina	1:500	Donkey anti‐chicken Cy3	Millipore	Retina	1:500
			Optic nerve				Optic nerve	
Glutamine synthetase	Abcam	Monoclonal	Retina	1:200	Goat anti‐mouse Alexa Fluor 488	Invitrogen	Retina	1:500
Phosphacan = 473HD epitope	Dep. of Cell Morphology & Mol. Neurobiology 90	Monoclonal	Retina	1:200	Goat anti‐rat Cy2	Dianova	Retina	1:250
			Optic nerve				Optic nerve	
Tenascin‐C	Dep. of Cell Morphology & Mol. Neurobiology 27	Polyclonal	Retina	1:200	Goat anti‐rabbit Cy3	Dianova	Retina	1:250
			Optic nerve				Optic nerve	
Vimentin	Sigma‐Aldrich	LN‐6	Retina	1:500	Donkey anti‐rabbit Alexa Fluor 488	Invitrogen	Retina	1:500

**Table 2:** Adjustments set for ImageJ macro. Before using the macro for area analysis of tenascin‐C, 473HD, GFAP, vimentin and glutamine synthetase, the background subtraction as well as the lower and the upper threshold must be set

Protein/epitope	Tissue	Background subtraction (pixel)	Lower threshold	Upper threshold
GFAP	Retina	100	19.49	201
Glutamine synthetase	Retina	20	7.95	80.5
Phosphacan = 473HD epitope	Retina	0	24.29	85
	Optic nerve	50	13.77	85
Tenascin‐C	Retina	0	28.24	85
	Optic nerve	50	13.03	85
Vimentin	Retina	100	20.16	247

### Macroglia cell analysis

To detect macroglia cells, retinae were stained with antibodies against glial fibrillary acidic protein (GFAP, astroglia), vimentin and glutamine synthetase (Müller glia) 14 days after immunization (*n* = 5/group). In addition, optic nerves were stained with an antibody against GFAP at day 14.

The retinal cross‐sections were incubated with 10% donkey and/or goat serum in 0.1% Triton‐X in PBS for 30–60 min. A mouse monoclonal anti‐GFAP, directly labelled with Alexa Fluor 488 (1:1.200; Millipore, Darmstadt, Germany), a mouse monoclonal anti‐vimentin antibody (1:500; Sigma‐Aldrich) and a mouse monoclonal anti‐glutamine synthetase antibody (1:200; Invitrogen) were applied overnight. The corresponding secondary antibodies goat antimouse Alexa Fluor 555 (1:400; Invitrogen) and goat antimouse Alexa Fluor 488 (1:500; Invitrogen) were added the next day for 60 min. Optic nerve sections were incubated with a chicken monoclonal GFAP antibody (1:500; Millipore) overnight. The secondary antibody, donkey anti‐chicken Cy3 (1:500; Millipore), was applied the next day for 60 min. Cell nuclei were visualized on retinal and optic nerve sections with DAPI. The photographs were taken using a fluorescence microscope (Axio Imager M1; Zeiss). Macroglia stainings were analyzed using an ImageJ macro, as describes above.

### Western blot

At 7, 14 and 28 days after immunization, retinae were used for Western blot analysis (*n* = 3–6/group). Proteins were isolated by mechanical and chemical methods. First, the frozen retinae were homogenized with a metal homogenizer (Neolab, Heidelberg, Germany), then 150 μl of lysis buffer (RIPA buffer; Cell Signaling Technology, Cambridge, UK) combined with protease inhibitory solution (Sigma‐Aldrich) was added. The retina solution was treated with ultrasound. Thereafter, the RIPA buffer was allowed to react on ice for 50 min. Remaining cell components were separated by centrifugation for 30 min. (4°C). The protein concentration was determined by a commercial bicinchoninic acid assay (BCA; Thermo Fisher Scientific). Twenty μg per sample was loaded on a lane of a 4–12% Bis‐Tris gel (NuPAGE; Invitrogen). After the blotting step using the NuPAGE Transfer buffer (60 min., 200 V), the nitrocellulose membranes were blocked with a mixture of 5% milk powder in a PBS/0.05% Tween‐20 solution. The primary antibodies anti‐tenascin‐C 27 (1:5000) and α‐tubulin (1:20,000; Sigma‐Aldrich) were used for the protein detection. The secondary antibodies were labelled with fluorochromes, like donkey anti‐rabbit Alexa Fluor 680 (1:5000; Invitrogen) and donkey anti‐mouse DyLight 800 (1:20,000; LI‐COR Bioscience, Lincoln, NE, USA). The protein bands were recorded and analyzed with the Odyssey infrared imager system 2.1 (LI‐COR Bioscience). The protein signal intensity was normalized to the reference protein α‐tubulin.

### RNA preparation and cDNA synthesis

For RNA isolation, retinae (*n* = 3–6/group) were prepared, stored in lysis buffer and snap frozen in liquid nitrogen 3, 7 and 14 days after immunization. Total retinal RNA was extracted using the Gene Elute Mammalian Total RNA Miniprep Kit according to the manufacturer's instructions. Optic nerve RNA (*n* = 3/group) was extracted using the ReliaPrepTM RNA Tissue Miniprep system (Promega, Madison, WI, USA) from snap frozen tissue 7 days after immunization. The quality and quantity of RNA were assessed by measurement of the ratio of absorbance values at 260 and 280 nm (BioSpectrometer^®^; Eppendorf, Hamburg, Germany). Total RNA (1 μg) was used for reverse transcription using cDNA synthesis kit (Thermo Fisher Scientific).

### Quantitative real‐time reverse transcription polymerase chain reaction

Primer sequences for all used primers were designed (Table 3). Quantitative real‐time PCR using SYBRGreen I (Roche Applied Science, Mannheim, Germany) technology was performed on the Light Cycler^®^ 96 (Roche Applied Science). Primer concentration was optimized to a final concentration of 200 nM and combined with 200 ng of retinal RNAs per well. We set‐up two reactions per sample RNA with a final volume of 20 μl per single reaction. The qRT‐PCR analyses were performed as described previously 29, 32. Each qRT‐PCR was performed at least in duplicates from retina and optic nerve tissue for each point in time and repeated two times. The average threshold cycle (Ct) values of the two independent experiments were used to calculate the ratios for the primers as described before 33. To obtain amplification efficiencies of different primer sets, standard curves by a twofold dilution series with template amounts ranging from 5 to 125 ng DNA per well were generated. The Ct values for the reference genes (retina: β*‐actin* and cyclophilin; optic nerve: cyclophilin) were used for normalization.

**Table 3:** Sequences of oligonucleotide pairs

Oligonucleotides	Sequences	Amplicon sizes (bp)
β*‐actin‐F*	cccgcgagtacaaccttct	72
β*‐actin‐R*	cgtcatccatggcgaact	
*Cyclophilin‐F*	tgctggaccaaacacaaatg	88
*Cyclophilin‐R*	cttcccaaagaccacatgct	
*GFAP‐F*	tttctccaacctccagatcc	64
*GFAP‐R*	gaggtggccttctgacacag	
*Glutamine synthetase‐F*	gaggcacagctgtaagcgtat	68
*Glutamine synthetase‐R*	cctgttccattccaaaccag	
*Pou4f1‐F*	ctccggaccttgagcttct	60
*Pou4f1‐R*	tagaagggagagttaaacacagaaca	
*RPTP*β*/*ζ*‐CA‐F*	aaccatccttggaaaacacg	66
*RPTP*β*/*ζ*‐CA‐R*	cattggtgagatttatttccactgt	
*RPTP*β*/*ζ*‐PTP1‐F*	cctcgtggagaaaggaagaag	77
*RPTP*β*/*ζ*‐PTP1‐R*	ccaggaagctcccgtattct	
*Tenascin‐C‐F*	gctctcctatggcatcaagg	60
*Tenascin‐C‐R*	tcatgtgtgaggtcgatggt	
*Vimentin‐F*	aacactcctgattaagacggttg	72
*Vimentin‐R*	tcatcgtggtgctgagaagt	

The primer pairs listed in the table were used in qRT‐PCR experiments. β*‐actin* and *cyclophilin* served as housekeeping genes for retinal samples. *cyclophilin* served as housekeeping gene in optic nerve samples. The predicted amplicon sizes are given.

F: forward; R: reverse.

### Statistical analysis

Immunohistology and Western blot data are presented as mean ± S.E.M., unless otherwise noted. The three groups were compared *via* anova followed by Tukey post‐hoc test using Statistica software (V12; Statsoft, Tulsa, OK, USA). For qRT‐PCR, statistical evaluation of Ct variations and calculated relative expression variations, data were analyzed for significant differences by a pairwise fixed reallocation and randomization test using REST^©^ program 33 and are shown as median ± quartile ± maximum/minimum. *P*‐values below 0.05 were considered statistically significant.

## Results

### No changes in RGC numbers

**Figure 1:**
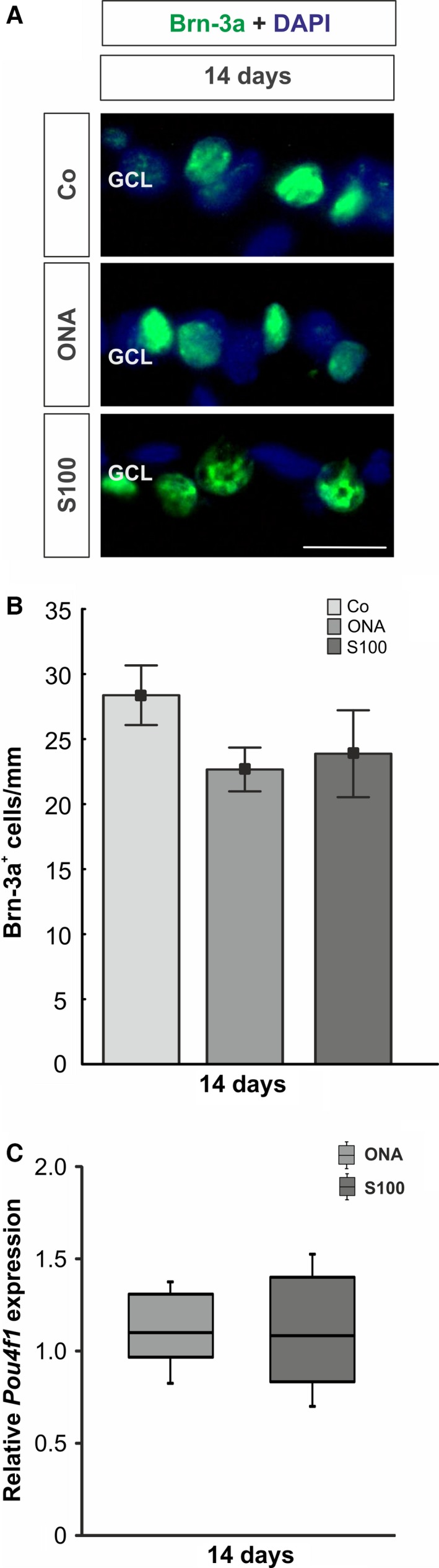
(**A**) The number of RGCs was evaluated *via* anti‐Brn‐3a staining (green) at 14 days. Cell nuclei were labelled with DAPI (blue). (**B**) The RGC count showed no differences between both immunized groups compared with the Co group (ONA:* P* = 0.3; S100: *P* = 0.4). (**C**) The mRNA expression of the RGC marker *Pou4f1* revealed no changes in either group (ONA:* P* = 0.4; S100: *P* = 0.6). Values for immunostaining are mean ± S.E.M. Values for qRT‐PCR are median ± quartile ± maximum/minimum. GCL: ganglion cell layer; scale bar: 20 μm.

It is known that a slow, but progressive RGC loss occurs in the EAG model. At about 28 days after immunization, a significant decline in RGCs can be detected in animals immunized with ONA and S100 4, 5, 34. To evaluate the number of RGCs at an earlier point in time, cross‐sections of the retina were stained with an anti‐Brn‐3a antibody after 14 days (Fig. 1A).

No alterations in the number of RGCs could be observed in the ONA group (22.66 ± 1.68 cells/mm, *P* = 0.3) compared with control group (28.37 ± 2.29 cells/mm; Fig. 1B). Also, no differences were noted in the S100 group (23.87 ± 3.34 cells/mm, *P* = 0.4). In addition, no alterations on mRNA level could be observed for *Pou4f1* (ONA: *P* = 0.4; S100: *P* = 0.6; Fig. 1C). As already mentioned above, the number of RGCs significantly decreases in both groups later on.

### Altered phosphacan expression in retina and optic nerve

**Figure 2:**
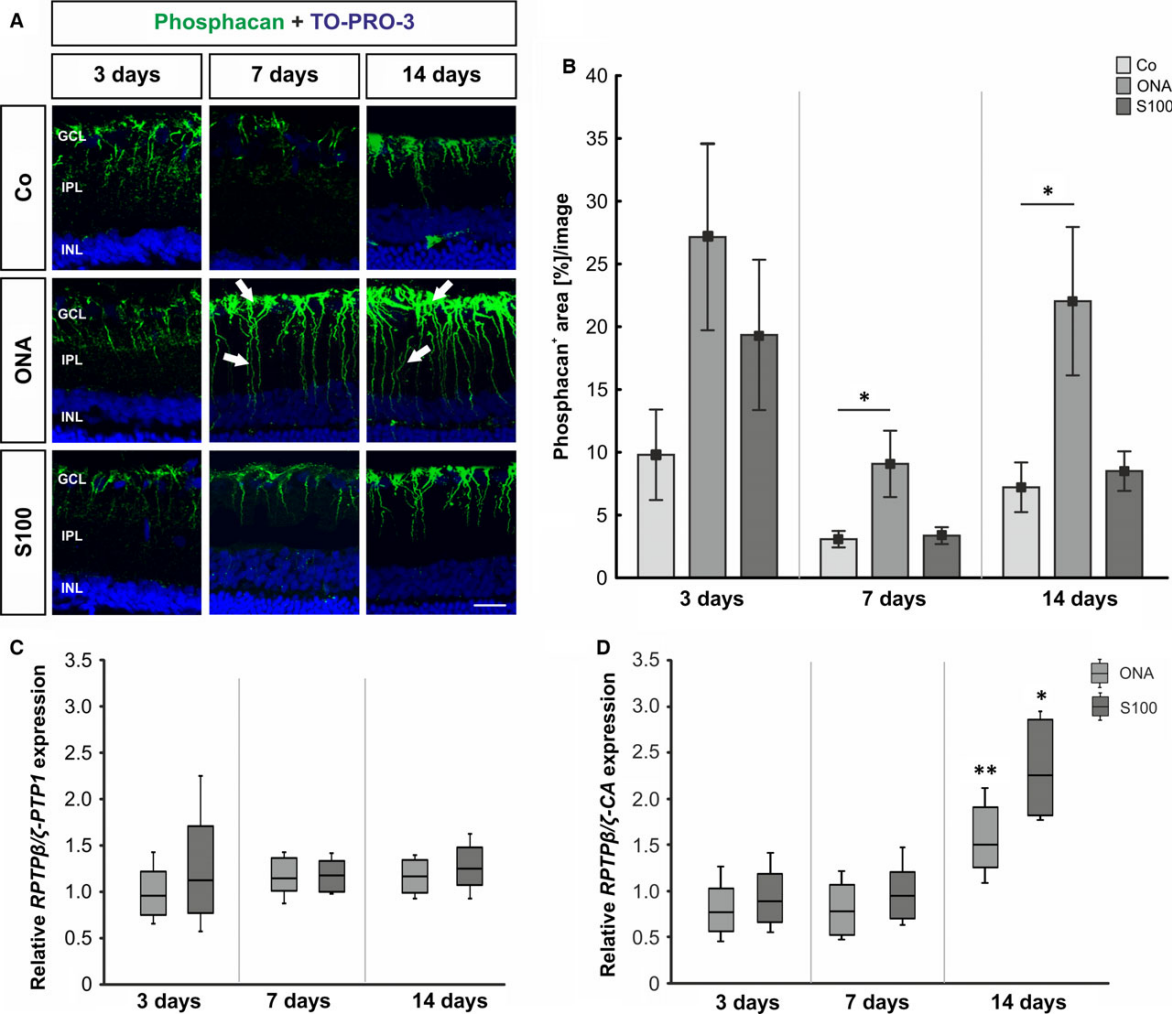
(**A**) Retinal cross‐sections were stained using the 473HD antibody (phosphacan, green) 3, 7 and 14 days after immunization. Cell nuclei were visualized with TO‐PRO‐3 (blue). The arrows point towards phosphacan labelling, mostly in the inner limiting membrane, with processes that span the retina. (**B**) No changes were observed with regard to phosphacan staining at 3 days in both immunized groups (*P* > 0.05). At 7 days, the phosphacan immunoreactivity was significantly higher in the ONA animals (*P* = 0.048), whereas the expression in the S100 retinae was not altered (*P* > 0.05). Phosphacan expression in ONA retinae was still increased (*P* = 0.048), whereas S100 animals were still not affected (*P* > 0.05) at 14 days. (**C**) At all points in time, no differences were found in the expression of the *RPTP*β*/*ζ receptor variants (*P* > 0.05), namely RPTPβ/ζ_long_ and RPTPβ/ζ_short_. (**D**) The mRNA levels of *RPTP*β*/*ζ*‐CA* revealed a significant increase in the ONA (*P* < 0.01) and the S100 group (*P* = 0.01) at 14 days. Values for immunostaining are mean ± S.E.M. and for qRT‐PCR median ± quartile ± maximum/minimum. GCL: ganglion cell layer; IPL: inner plexiform layer; INL: inner nuclear layer; phosphacan: 473HD‐epitope; scale bar: 20 μm; * *P* < 0.05, ** *P* < 0.01.

**Figure 3:**
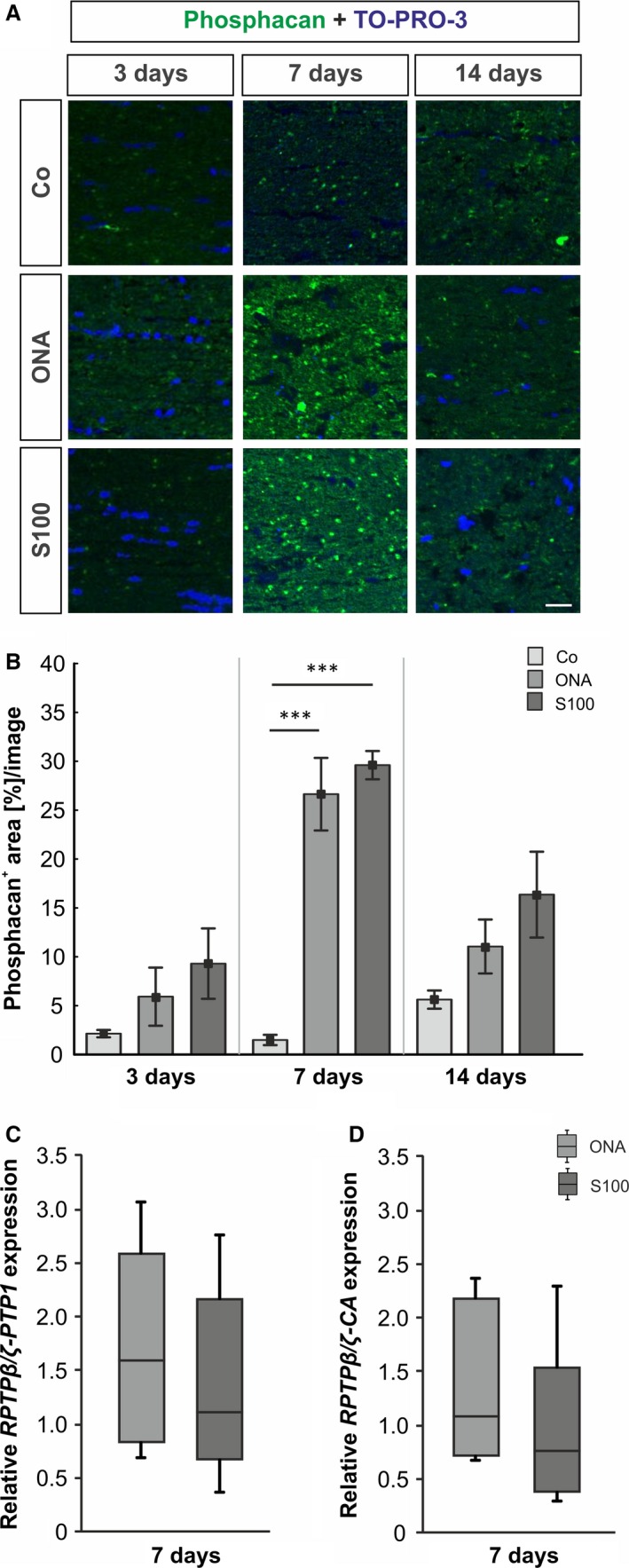
(**A**) Optic nerves were stained using the anti‐473HD antibody (phosphacan, green) and TO‐PRO‐3 (cell nuclei, blue) at all points in time. (**B**) At 3 days, no changes in the 473HD staining could be observed in both immunized groups (*P* > 0.05). Strong phosphacan immunoreactivity was noted in ONA and S100 retinae 7 days after immunization (ONA:* P* = 0.0002; S100: *P* = 0.0002). At 14 days, phosphacan expression went back to control levels in both immunized groups (*P* > 0.05). (**C**) The mRNA levels of *RPTP*β*/*ζ*‐PTP1* and *RPTP*β*/*ζ*‐CA* revealed no changes in the ONA and the S100 group (*P* > 0.05) 7 days post‐immunization. Values are mean ± S.E.M. and for qRT‐PCR median ± quartile ± maximum/minimum. Phosphacan: 473HD‐epitope; scale bar: 40 μm; *** *P* < 0.001.

To investigate the immunoreactivity of the 473HD‐epitope, namely phosphacan, sections of the retina and the optic nerve were stained using the 473HD antibody 3, 7, 14 and 28 days after immunization (Fig. 2A and 3A, and Fig. S1A and B). Moreover, to analyze the expression level of RPTPβ/ζ isoforms in the retina, we performed qRT‐PCR analyses of the carbonic anhydrase‐like domain of all three RPTPβ/ζ isoforms (*RPTP*β*/*ζ*‐CA*) as well as for the protein tyrosine phosphatase domain of the receptor‐type isoforms *RPTP*β*/*ζ_*long*_ and *RPTP*β*/*ζ_*short*_ (RPTPβ/ζ‐PTP1) 3, 7 and 14 days after immunization (Fig. 2C and D).

In the retina, 3 days after immunization, no changes in the phosphacan immunoreactivity could be noted in both immunized groups (ONA: 27.15 ± 7.48%/image, *P* = 0.1; S100: 19.35 ± 5.99%/image, *P* = 0.5, Fig. 2B) compared with the control group (9.80 ± 3.61%/image). At 7 days, phosphacan staining increased in the ONA animals (Co: 3.08 ± 0.66%/image; ONA: 9.08 ± 2.66%/image, *P* = 0.048), whereas no alterations were observed in the S100 group (3.37 ± 0.67%/image, *P* = 0.9). The staining of phosphacan was still increased in the ONA group at 14 days (Co: 7.21 ± 1.98%/image, ONA: 22.04 ± 5.91%/image, *P* = 0.048), whereas it was still not altered in the S100 group (8.50 ± 1.58%/image, *P* = 0.9). After 28 days, no changes in the phosphacan immunoreactivity was observed in the ONA group (*P* = 0.9) and in the S100 group (*P* = 0.2; Fig. S1A and C).

With regard to the mRNA level of *RPTP*β*/*ζ_*lo*ng_ and RPTP‐β/ζ_short_, detected by using *RPTP*β*/*ζ*‐PTP1* oligonucleotides, no changes in the expression could be noted in both groups, ONA and S100, at all points in time (*P* > 0.05; Fig. 2C). The expression of all three *RPTP*β*/*ζ isoforms, as verified by using the *RPTP*β*/*ζ*‐CA* oligonucleotides, did not altered at 3 and 7 days (*P* > 0.05). But a significant up‐regulation was found in the ONA group (1.5‐fold, *P* = 0.005) as well as in the S100 group (2.25‐fold, *P* = 0.02) at 14 days after immunization (Fig. 2D). This finding implied that the secreted isoform phosphacan, instead of the *RPTP*β*/*ζ receptor variants, is up‐regulated at 14 days in both immunized groups.

In the optic nerve, no altered phosphacan immunoreactivity could be observed in both immunized groups at 3 days (Co: 2.14 ± 0.38%/image; ONA: 5.92 ± 2.97%/image, *P* = 0.6; S100: 9.30 ± 3.60%/image, *P* = 0.3; Fig. 3B). Then, after 7 days, the phosphacan immunoreactivity was increased in the ONA groups (Co: 1.49 ± 0.52%/image; ONA: 26.64 ± 3.71%/image, *P* = 0.0002) as well as in the S100 group (29.61 ± 1.45%/image, *P* = 0.0002). After 14 days, phosphacan staining in the ONA group (11.06 ± 2.76%/image, *P* = 0.4) and in the S100 group (16.36 ± 4.39%/image, *P* = 0.08) went back closer to the control level (5.63 ± 0.93%/image). Also, 28 days post‐immunization no changes were observed in the ONA group (*P* = 0.9) and the S100 group (*P* = 0.8; Fig. S1B and D). No alterations in the expression of *RPTP*β*/*ζ*‐PTP1* receptor variants were noted in the ONA (*P* = 0.3) and in the S100 group (*P* = 0.8) 7 days after immunization (Fig. 3C). Also, no changes were observed in the *RPTP*β*/*ζ*‐CA* expression in either groups (ONA: *P* = 0.9; S100: *P* = 0.5; Fig. 3D).

### Early increase in tenascin‐C expression in retina and optic nerve tissue

**Figure 4:**
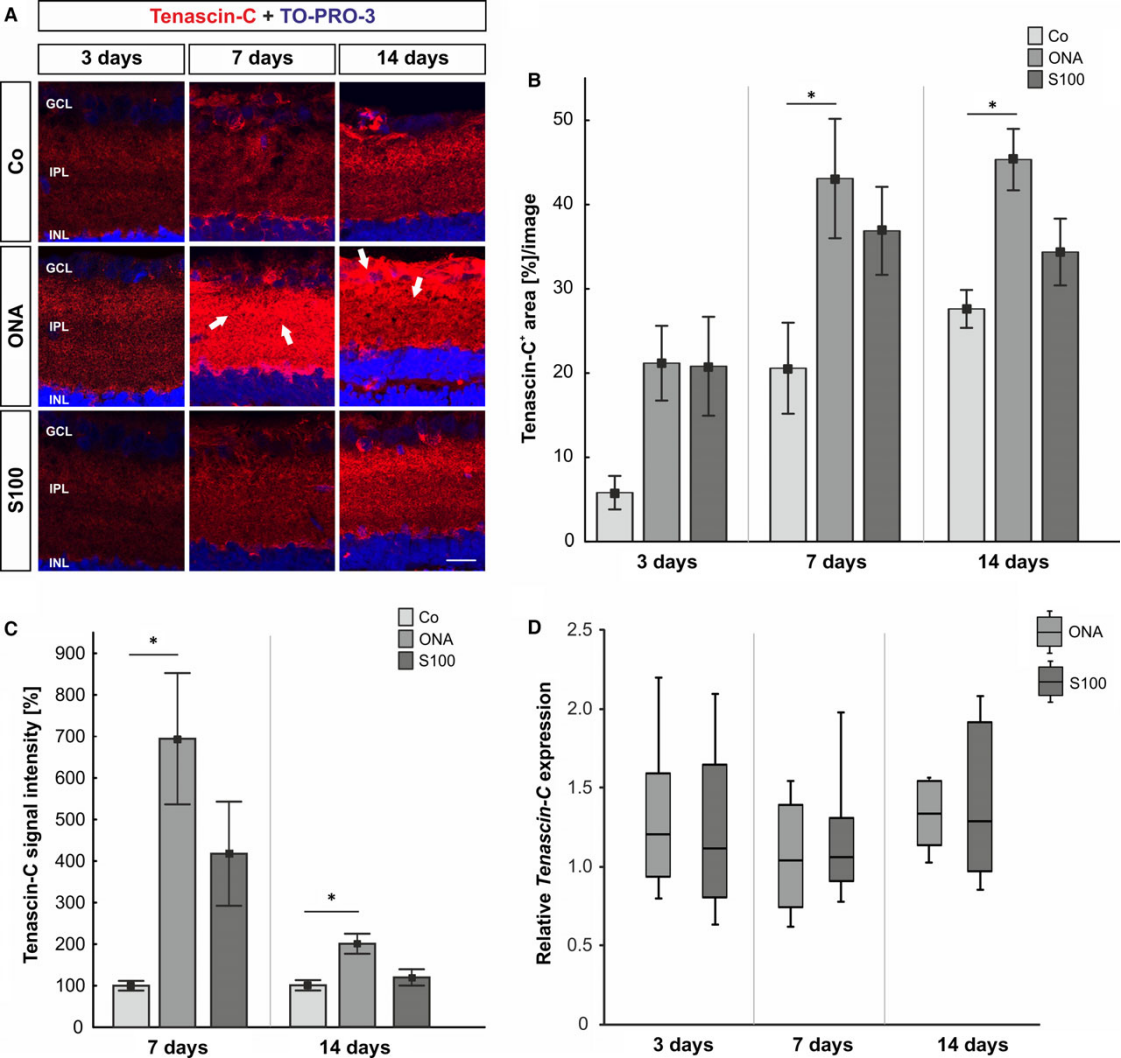
(**A**) At 3, 7 and 14 days retinal cross‐sections were stained with an anti‐tenascin‐C antibody (red). At 7 days, the staining is more consistent in the inner retina (arrows). An increased immunoreactivity of tenascin‐C was seen in the ganglion cell layer and inner plexiform layer (arrows). (**B**) At 3 days, no alterations were noted in either immunized groups (*P* > 0.05). After 7 and 14 days, tenascin‐C reactivity was significantly higher in the ONA groups (7 days: *P* = 0.04; 14 days: *P* = 0.01), whereas the S100 group was not affected (*P* > 0.05). (**C**) The protein level of tenascin‐C showed an increase in the ONA group (7 and 14 days: *P* = 0.02), whereas the S100 group was not affected (7 days: *P* = 0.2; 14 days: *P* = 0.8). (**D**) The mRNA expression levels of *tenascin‐C* revealed no changes at all points in time (*P* > 0.05). Values for immunostaining and Western blot are mean ± S.E.M. Values for qRT‐PCR are median ± quartile ± maximum/minimum. GCL: ganglion cell layer; IPL: inner plexiform layer; INL: inner nuclear layer; scale bar: 20 μm; * *P* < 0.05.

**Figure 5:**
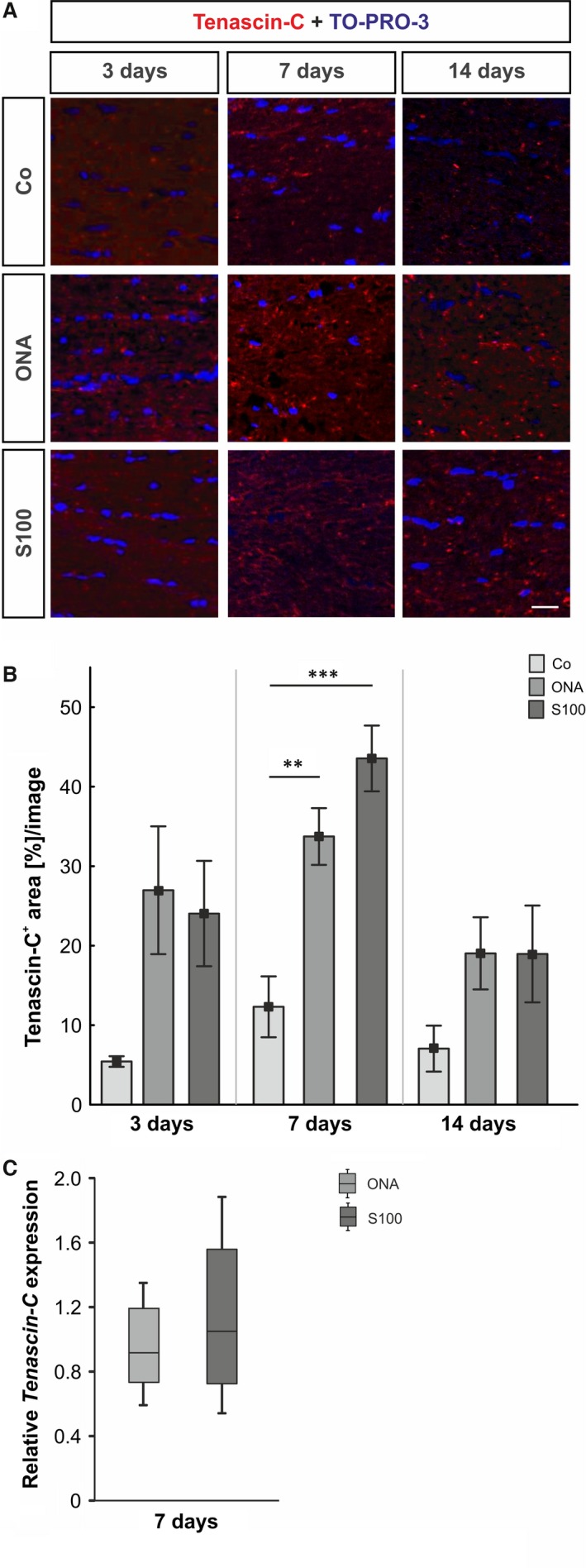
(**A**) Optic nerves were stained with an anti‐tenascin‐C antibody (red) at 3, 7 and 14 days. Cell nuclei were visualized with TO‐PRO‐3 (blue). (**B**) The immunoreactivity of tenascin‐C did not change in both groups after 3 days (*P* > 0.05). At 7 days, an up‐regulation of tenascin‐C was noted in the ONA group (*P* = 0.009) and in the S100 group (*P* = 0.0009). No alterations could be noted in both groups after 14 days (*P* > 0.05). (**C**) The expression of the *tenascin‐C* mRNA revealed no changes in either group at day 7 (*P* > 0.05). Values for immunostaining are mean ± S.E.M. and for qRT‐PCR median ± quartile ± maximum/minimum; scale bar: 40 μm; ** *P* < 0.01, *** *P* < 0.001.

To evaluate the immunoreactivity of the glycoprotein tenascin‐C, retina and optic nerve sections were labelled with an anti‐tenascin‐C antibody at days 3, 7, 14 and 28 (Fig. 4A and 5A, and Fig. S2A and B). Western blot analysis of the retinae was performed at 7, 14 and 28 days (Fig. 4C). Retinae were also used for qRT‐PCR expression analysis of tenascin‐C 3, 7 and 14 days after immunization (Fig. 4D). In addition, qRT‐PCR analyses were performed from the optic nerves 7 days post‐immunization (Fig. 5C).

The immunohistochemistry of retinas data revealed no changes in tenascin‐C staining in both immunized groups at 3 days (Co: 5.81 ± 1.99%/image; ONA: 21.18 ± 4.44, *P* = 0.07; S100: 20.81 ± 5.87%/image, *P* = 0.08, Fig. 4B). At 7 days, significantly more tenascin‐C immunoreactivity was noted in the ONA group (Co: 20.58 ± 5.44%/image; ONA: 43.09 ± 7.09%/image, *P* = 0.04), whereas no significant alterations could be observed in the S100 group (36.88 ± 5.22%/image, *P* = 0.2). In the ONA group an increased tenascin‐C staining area was noted after 14 days (Co: 27.61 ± 2.25%/image; ONA: 43.34 ± 3.65%/image, *P* = 0.01), whereas the S100 group was still not affected (34.37 ± 3.96%/image, *P* = 0.4). At 28 days, no alterations were noted in the ONA group (*P* = 0.8) compared with the controls. Also, no changes were observed in the S100 group (*P* = 0.9) at this point in time (Fig. S2A). *Via* Western blot, a significant up‐regulation of tenascin‐C was noted in the ONA group (*P* = 0.02) after 7 days. No changes were observed in the S100 group (*P* = 0.2). Also at 14 days, the protein level of tenascin‐C was significantly up‐regulated in the ONA group (*P* = 0.02), whereas the level in the S100 group was not affected (*P* = 0.8). 28 days post‐immunization, no alterations were noted (ONA: *P* = 0.8; S100: *P* = 0.08, Fig. 4C). Analyses of the relative *Tenascin‐C* mRNA expression levels revealed no changes in either group at 3, 7 and 14 days (*P* > 0.05; Fig. 4D).

With regard to the optic nerves, the immunoreactivity of tenascin‐C did alter neither in the ONA group (26.98 ± 8.03%/image, *P* = 0.06) nor in the S100 group (24.04 ± 6.62%/image, *P* = 0.1) compared with the controls (5.43 ± 0.67%/image; Fig. 5B) at 3 days. At 7 days, an up‐regulation of tenascin‐C was noted in the ONA group (Co: 12.31 ± 3.83%/image; ONA: 33.73 ± 3.57%/image, *P* = 0.009) and also in the S100 group (43.55 ± 4.14%/image, *P* = 0.0009). No significant changes in the tenascin‐C staining were noted in the S100 (18.96 ± 6.09%/image, *P* = 0.2) and ONA group (19.03 ± 4.54%/image, *P* = 0.2) compared with controls (7.05 ± 2.89%/image) 14 days post‐immunization. Also, after 28 days, no differences in the immunoreactivity of tenascin‐C were observed in the ONA group (*P* = 0.9) and the S100 group (*P* = 0.3; Fig. S2D). The qRT‐PCR analyses revealed no changes in the *tenascin‐c* expression in the ONA (*P* = 0.6) and in the S100 group (*P* = 0.9) at 7 days (Fig. 5C).

### Little macroglia response in the retina and optic nerve

**Figure 6:**
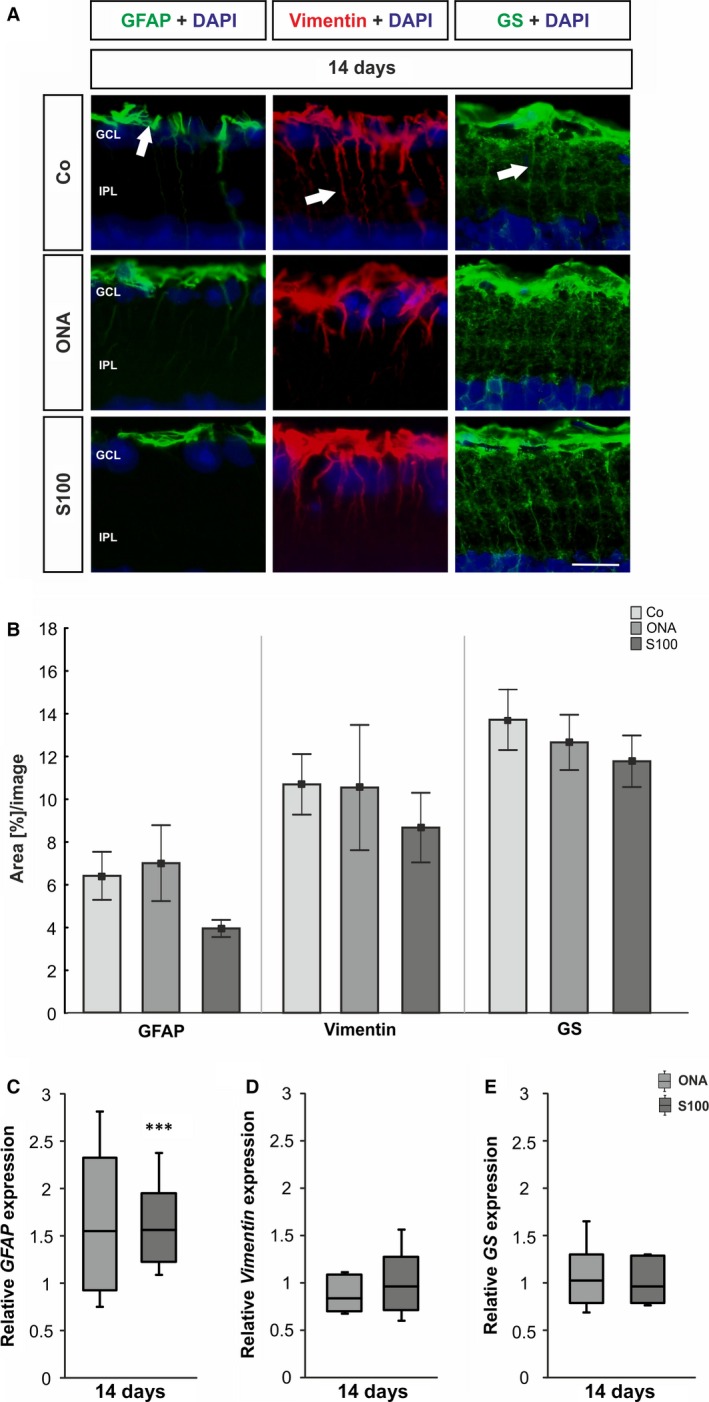
(**A**) Staining of retinal cross‐sections with anti‐GFAP (green), anti‐vimentin (red), anti‐glutamine synthetase (green) and DAPI (cell nuclei, blue) 14 days after immunzation. In the control group GFAP is mostly expressed in the nerve fibre and ganglion cell layer (arrow). Vimentin and glutamine synthetase seem to label mainly Müller cells in the retina (arrows). (**B**) The GFAP ^+^ area analysis revealed no alterations in both immunized groups (*P* > 0.05). No changes in vimentin and glutamine synthetase staining could be observed for the ONA and the S100 group at 14 days (*P* > 0.05). (**C**) The qRT‐PCR revealed an increase in *GFAP* expression in the S100 group (p < 0.001), whereas the ONA group was not affected (*P* > 0.05). (**D**) For *vimentin* expression, no alterations were observed in either groups (*P* > 0.05). (**E**) Also, the expression of *glutamine synthetase* was not altered in the ONA and S100 group (*P* > 0.05). Values for immunostaining are mean ± S.E.M. and for qRT‐PCR median ± quartile ± maximum/minimum. GCL: ganglion cell layer; IPL: inner plexiform layer; GS: glutamine synthetase; scale bar: 20 μm; *** *P* < 0.001.

To detect possible alterations in the macroglia cells, cross‐sections of the retinae were stained with an anti‐GFAP, an anti‐vimentin and an anti‐glutamine synthetase antibody (Fig. 6A). Also, qRT‐PCR analyses of *GFAP*,* vimentin* and *glutamine synthetase* were performed after 14 days (Fig. 66C,D,E). Vimentin and glutamine synthetase are preferentially expressed by Müller glia, the anti‐GFAP antibody marks mainly astrocytes. In addition, sections of the optic nerve were stained with an anti‐GFAP antibody 14 days after immunization. Also, analyses of the *GFAP* expression were performed *via* qRT‐PCR at this point in time.

For GFAP immunoreactivity in the retinae, no alterations could be observed in the ONA group (6.99 ± 1.77%/image, *P* = 0.9) compared with the control group (6.39 ± 1.12%/image; Fig. 6B). Additionally, no alterations of GFAP immunostaining were noted in the S100 group (3.93 ± 0.40%/image, *P* = 0.4). But, an up‐regulated *GFAP* mRNA expression was observed (*P* < 0.001; Fig. 6C). No changes in *GFAP* mRNA expression could be noted in the ONA group (*P* = 0.2).

Vimentin staining was comparable in the ONA (10.40 ± 2.98%/image, *P* = 0.9), the S100 (8.56 ± 1.71%/image, *P* = 0.8) and the control group (10.52 ± 1.15%/image) at 14 days (Fig. 6B). The analysis of *vimentin* mRNA expression showed no alterations in either group (ONA: *P* = 0.4; S100: *P* = 0.8; Fig. 6D).

Regarding the glutamine synthetase staining, no alterations were noted in the ONA group (13.29 ± 1.36%/image, *P* = 0.8) compared with the control group (14.41 ± 1.49%/image). Also, no changes were observed in the S100 group (12.37 ± 1.27%/image, *P* = 0.6) 14 days after immunization (Fig 6B). Evaluation of the *glutamine synthetase* mRNA revealed changes neither in the ONA nor in the S100 group (*P* > 0.05, Fig. 6E).

In the optic nerves, the GFAP staining in the Co group showed homogenous and less ramified GFAP signal. In the ONA and S100 group, the GFAP signal was more disorganized (Fig. S3A). The qRT‐PCR analyses revealed no changes in the *GFAP* expression in the ONA group (*P* = 0.1), whereas a significant up‐regulation was noted in the optic nerves of the S100 group (*P* = 0.03; Fig. S3B).

## Discussion

The causes for glaucoma are still poorly understood. An elevated IOP is the main risk factor for glaucoma. In the last years, several possible pathogenic factors, like ischemic 35, 36 and immunological mechanisms 2, 37, or oxidative stress 38, 39, were discussed. Oxidative stress is an imbalance between the processes that generate reactive oxygen species (ROS) and those responsible for their removal. It damages macromolecules including DNA and proteins and is thought to contribute to the pathogenesis of neurodegenerative diseases, like Morbus Alzheimer's and Parkinson's disease 40. Oxidative stress might result in indirect or direct damage to the RGCs. Indirectly, the damage could occur through aberrant immune response or glial dysfunction. It also triggers RGC apoptosis, which could be observed *in vitro* 41, 42. In animal models with and without elevated IOP, a relation between oxidative stress and RGC loss was found 43, 44. Similar results could also be observed in humans with primary open‐angle and normal tension glaucoma 45, 46, 47, 48. But it should be stated that until now, it remains unclear whether oxidative stress is a risk factor for glaucoma or rather an epiphenomenon 49.

In this study, we analyzed the expression pattern of the ECM glycoprotein tenascin‐C and the CSPG phosphacan/RPTPβ/ζ in an IOP‐independent EAG model. Data from previous studies showed a loss of RGCs in the ONA as well as in the S100 group 28 days after immunization 4, 5. Here, we investigated possible ECM alterations in this model at earlier points in time. One of the most interesting observation of this study was that ECM remodelling occurred shortly after immunization, before reactive gliosis and RGC loss are present. Based on our findings, we speculate that tenascin‐C and phosphacan might act as early indicators of retinal neurodegeneration.

The role of matricellular molecules in glaucoma disease is emerging as some have a close association with pathological events in the TM as well as in the lamina cribrosa 10, 50, 51. In high‐tension glaucoma, elevated IOP leads to mechanical stretching of TM cells, which induces ECM remodelling 52. Tenascin‐C and other ECM components, including fibronectin, periostin and collagens type I and V, were reported to act as important modulators of connective tissue by regulating the assembly of collagen fibrils, namely fibrosis, to impact the biomechanical properties 53, 54. This might reflect a possible mechanism by which connective tissue withstands tensile forces in glaucoma 10. Indeed, after IOP‐induced mechanical stretching of the TM, tenascin‐C was found to be up‐regulated. Nevertheless, tenascin‐C knockdown was not suggested to contribute to outflow resistance 55. Although tenascin‐C knockdown does not directly contribute to TM outflow resistance, its localization in the juxtacanalicular region suggests that TM regions are continuously remodelled without and with IOP elevation to control tissue homeostasis 55, 56. Because of reports of tenascin‐C remodelling in the TM, we speculate that in our study the remodelling in the retina and optic nerve is a result of this imbalanced homeostasis. In this context, IOP elevation and mechanical stretching also influence alternative splicing events that lead to the modulation of potential glycosaminoglycan attachment sites and additional ECM‐binding motifs 52. Also, GAGs were reported to implicate the regulation of aqueous humor outflow resistance through the TM 57. In addition, MMPs contribute to ECM remodelling in glaucomatous tissue 58.

In the developing CNS, the hexameric tenascin‐C glycoprotein is *highly* expressed. With ongoing maturation of the CNS until adulthood, tenascin‐C is progressively down‐regulated, but is re‐expressed under pathological conditions when neurodegeneration, infection, inflammation or damage occurs 7, 59. Tenascin‐C is a high‐affinity ligand for phosphacan and both exhibit prominent colocalization, especially during CNS development 13, 60. In the retina, the overlapping expression of both also suggests an interaction between the molecules. Because of the widely accepted concept that CSPGs act permissively for axonal growth during development and also during regeneration processes, it was speculated that phosphacan acts as boundary formation molecule for retinal axons 7, 61, 62. Hence, phosphacan has been described to inhibit the growth of RGCs *in vitro* 63.

In the adult retina, tenascin‐C is expressed by amacrine and displaced amacrine cells as well as by horizontal cells 64. In central nervous tissue a dynamic regulation of tenascin‐C was additionally described in reactive astrocytes after lesions 65, 66, 67, 68. Based on these findings, we speculate that reactive astrocytes of the optic nerve and neuronal cells of the retina up‐regulate tenascin‐C following RGC death and axon degeneration.

As demonstrated by Pena *et al*. 8, tenascin‐C expression levels are highly associated with reactive astrocytes in the glaucomatous optic nerve. Although the precise role of tenascin‐C in glaucoma is not yet elucidated, an enhanced expression might act protectively on RGC axons 8. On the other hand, changes in glial functions in glaucoma are often accompanied by dramatic alterations in the synthesis of ECM 69, 70. ECM remodelling may lead to additional damage of RGC axons. Also, age‐related alterations in glial ECM synthesis have been proposed to increase the susceptibility of glaucomatous damage 38. Recently, it was shown by Vecino *et al*. that the ECM environment influences regeneration and survival of adult RGC subtypes as result of a different integrin composition 71.

In previous studies of our group we could demonstrate that apoptotic processes occur in the retina as early as 14–22 days after ONA immunization 6, which might induce tenascin‐C remodelling. Also, optic nerve degeneration was noted in the EAG model at days 14 and 28 34, 72. Other studies have already demonstrated that tenascin‐C expression is highly associated with glial scar formation. It was speculated that tenascin‐C displays barrier function to confine hurtful influences. Therefore, it might also act as a neuroprotective molecule in retinal neurodegeneration. Studies also indicate that ECM components act as important immune modulators. Increased levels of pro‐inflammatory cytokines, including tumour necrosis factor‐α (TNF‐α), can be detected during glaucoma neurodegeneration 73, 74. These might then influence the expression of matrix components. Notably, tenascin‐C seems to play an important immune‐modulatory role 75, including influencing the immune system *via* toll‐like receptor‐4 and is regulated by cytokines during inflammation 23. Moreover, the transforming growth factor‐β (TGF‐β) stimulates tenascin‐C expression 76. In the optic nerve, TGF‐β‐induced ECM changes were correlated with an impaired axonal transport and neurotrophic supply that lead to a continuous degeneration of axons 77. Astrocytes of the optic nerve head represent a main source of TGF‐β and the tenascin‐C glycoprotein. Interestingly, soluble TNF‐α was recently identified as important modulator of synaptic plasticity by its ability to increase Ca^2+^‐permeable α‐amino‐3‐hydroxy‐5‐methyl‐4‐isoxazolepropionic acid receptor expression, which directly contributes to RGCs death in an ocular hypertension glaucoma animal model 73. Also, in other retinal degeneration models, for example a diabetic retinopathy model, neurodegeneration is accompanied by increased levels of pro‐inflammatory cytokines, such as interleukin‐β and TNF‐α 78. Interestingly, both inflammatory mediators as well as the increased production of ROS were described to alter the production of several ECM proteins, including tenascin‐C.

An activation of the innate immune system, especially of the complement system, was also observed in the EAG model. Here, the activation also took place at an early point in time after immunization and before RGC death occurred 79. A higher tenascin‐C expression was also described to be associated with chronic inflammation in autoimmune diseases 80. Based on these findings, tenascin‐C might promote or restrict neurodegeneration in glaucoma, depending on the point in time of neurodegeneration, the cellular content as well as the surrounding cellular microenvironment.

Regarding glaucoma, an up‐regulation of tenascin‐C was previously shown in an ocular hypertension animal model 9. Moreover, reactive astrocytes showed an increased expression in the optic nerve of glaucoma patients with IOP elevation 8. It was assumed that the remodelling of tenascin‐C conducted axonal stability during IOP elevation 81. In this study, it was shown that tenascin‐C remodelling is evident in an IOP‐independent model. Therefore, IOP elevation seems to be not the initiating factor, in contrast to retinal damage.

The DSD‐1‐PG, recognized by the 473HD antibody, represents a homologue of the CSPG Phosphacan/RPTPβ/ζ. CSPGs act mainly as inhibitory molecules during retinal regeneration as well as in the TM of human glaucomatous eyes 82, 83. They can induce immune responses 84 and inhibit regenerative processes in the CNS 85. In an experimental autoimmune uveitis model, degradation of CSPGs exerts beneficial effects on neuronal survival 86. Our previous studies demonstrated that the 473HD epitope is restricted to Müller glia of the retina. In the optic nerve, prominent 473HD immunoreactivity seems to be associated with astrocytes 7, 87. Although only few changes in the mRNA expression of *GFAP* were noted in this study after 14 days. Previous results of our group revealed a reactive gliosis at later points in time 4. Here, phosphacan expression in Müller glia occurs earlier following retinal and optic nerve damage than gliosis in this model. Another CSPG, neurocan, was found to be up‐regulated in the retina after transient ischaemia 88. Moreover, in a retinal laser lesion model *phosphacan/RPTP*β*/*ζ exhibits increased expression levels 66. It was shown that it promotes axonal regeneration and repair processes in the CNS 89. As a result of these findings, phosphacan might also act as a neuroprotective molecule in this IOP‐independent model.

In the CNS, phosphacan/RPTPβ/ζ interacts directly with a variety of glycoproteins, including tenascin‐C 19, 20. It also interacts with other cell surface components and growth factors 11. Interestingly, both molecules were found to be up‐regulated simultaneously in the immunized retina and optic nerve. Although both molecules display expression by different cellular identities, namely tenascin‐C in amacrine cells and astrocytes and phosphacan in Müller glia, it is tempting to speculate that both ECM molecules interact with each other to reduce early retinal damage in this model.

In the work presented here, we demonstrated an early remodelling of ECM proteins after immunization with ocular antigens. The immunoreactivity of tenascin‐C and phosphacan/RPTPβ/ζ was up‐regulated already after 7 days, before RGC loss and gliosis occurred in this model. Interestingly, the increased expression of both proteins started simultaneously in retina and optic nerve. The alteration of both ECM proteins might act as an early indicator for glaucoma disease.

## Conflict of interest

The authors confirm that there are no conflicts of interest.

## Supporting information


**Figure S1** (**A**) Sections of retinae stained with an 473HD antibody (phosphacan, green) and TO‐PRO‐3 (blue) 28 days after immunization. (**C**) No changes were observed in the retinae with regard to phosphacan staining in the ONA and S100 group (*P* > 0.05). (**B**) Optic nerves were also labelled with an 473HD (phosphacan) antibody (green). Cell nuclei were stained with TO‐PRO‐3 (blue) at day 28. (**D**) Area analyses revealed no alterations in either immunized groups with regard to the phosphacan staining (*P* > 0.05). Values are mean ± S.E.M. GCL: ganglion cell layer; IPL: inner plexiform layer; INL: inner nuclear layer; phosphacan: 473HD‐epitope; scale bar in **A**: 20 μm, in **B**: 40 μm.

**Figure S2** (**A**) Retinal cross‐sections were labelled with an antibody against tenascin‐C (red) and TO‐PRO‐3 (blue) after 28 days. (**C**) The immunohistology showed no differences in the ONA and S100 group compared with the controls (*P* > 0.05). (**E**) Also, the Western blot experiments revealed no changes in either groups at day 28 (*P* > 0.05). (**B**) Optic nerves were stained with an anti‐tenascin‐C antibody (red) at 28 days. (**D**) The immunoreactivity of tenascin‐C did not change in the ONA and in the S100 group at this point in time (*P* > 0.05). Values are mean ± S.E.M. GCL: ganglion cell layer; IPL: inner plexiform layer; INL: inner nuclear layer; scale bar in **A**: 20 μm, in **B**: 40 μm.

**Figure S3** (**A**) Representative optic nerve photos of the Co, ONA and S100 group labelled with an anti‐GFAP antibody (red) 14 days after immunization. Cell nuclei were visualized with DAPI (blue). In the Co group, a homogenous and less ramified GFAP signal could be observed. In the ONA and S100 group, GFAP labelling was more disorganized. (**B**) The expression level of *GFAP* revealed no changes in the ONA group (*P* > 0.05), whereas a significant up‐regulation was noted in the S100 group (*P* = 0.03) after 14 days. Values are median ± quartile ± maximum/minimum; scale bar: 20 μm.
