# Supplementary figures and images for: Early remodelling of the extracellular matrix proteins tenascin‐C and phosphacan in retina and optic nerve of an experimental autoimmune glaucoma model
Source: J Cell Mol Med. 2016 Jul 4;20(11):2122–37. doi: 10.1111/jcmm.12909 (PMC5082392; doi:10.1111/jcmm.12909)

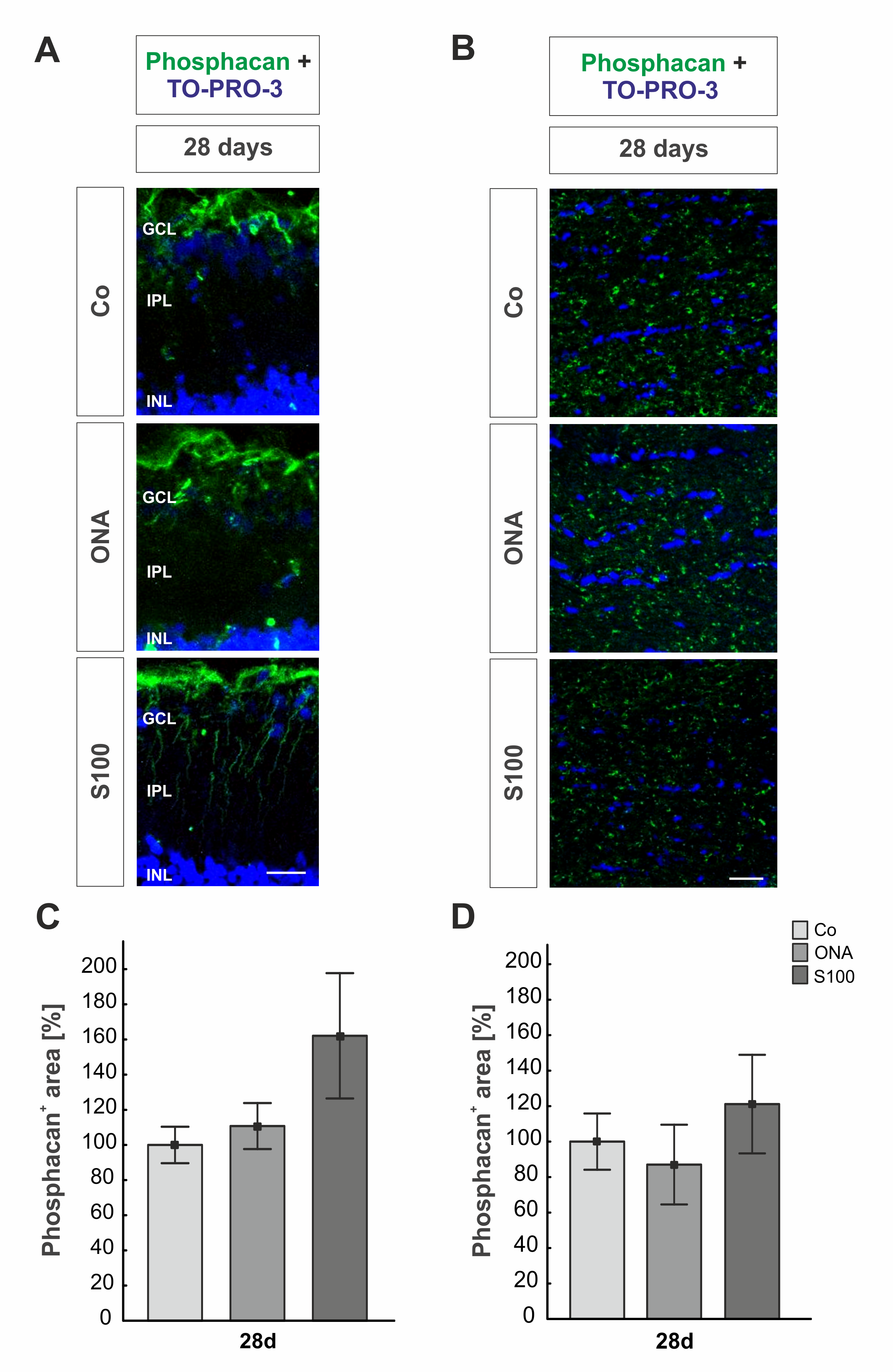

Supplement: Supplementary file 1 — Figure S1 (A) Sections of retinae stained with an 473HD antibody (phosphacan, green) and TO‐PRO‐3 (blue) 28 days after immunization. (C) No changes were observed in the retinae with regard to phosphacan staining in the ONA and S100 group (P > 0.05). (B) Optic nerves were also labelled with an 473HD (phosphacan) antibody (green). Cell nuclei were stained with TO‐PRO‐3 (blue) at day 28. (D) Area analyses revealed no alterations in either immunized groups with regard to the phosphacan staining (P > 0.05). Values are mean ± S.E.M. GCL: ganglion cell layer; IPL: inner plexiform layer; INL: inner nuclear layer; phosphacan: 473HD‐epitope; scale bar in A: 20 μm, in B: 40 μm. [file JCMM-20-2122-s001.tif]
